# Isolation and Characterization of Two Monoterpene Synthases and a Sesquiterpene Synthase from *Asarum heterotropoides*

**DOI:** 10.3390/metabo15110753

**Published:** 2025-11-20

**Authors:** Jiayi Li, Qianhua Shen, Yongze Zhang, Hanshu Tao, Bingyi Xu, Xiaoyan Min, Haiyang Liu, Na Han, Xin Fang

**Affiliations:** 1 School of Traditional Chinese Materia Medica, Shenyang Pharmaceutical University, Shenyang 110016, Chinaxiaoyanmin123@126.com (X.M.); 2 Key Laboratory of Phytochemistry and Natural Medicines, Kunming Institute of Botany, Chinese Academy of Sciences, Kunming 650201, China; haiyangliu@mail.kib.ac.cn

**Keywords:** terpenoids, monoterpene synthase, sesquiterpene synthase, promiscuous enzyme, *Asarum heterotropoides*, essential oil

## Abstract

**Background**: *Asarum heterotropoides*, a prominent medicinal plant in China, is well known for producing an abundance of monoterpenes and sesquiterpenes, which constitute the primary components of its essential oil and serve as the principal active compounds of the species. However, the biosynthetic pathways for these terpenoids remain largely unelucidated. **Methods**: Gas chromatography–mass spectrometry analysis, in vitro enzyme assay, subcellular localization experiment and molecular docking were used to characterize the function of terpene synthase from *A. heterotropoides*. **Results**: In this study, we isolated and characterized two monoterpene synthases and one sesquiterpene synthase from *A. heterotropoides*. These enzymes exhibit promiscuous activities, accepting geranyl pyrophosphate and farnesyl pyrophosphate as substrates to yield a variety of monoterpene and sesquiterpene products in in vitro enzymatic assays. All three enzymes possess a conserved RRx8W motif, a hallmark typically associated with TPS-b and TPS-d monoterpene synthases involved in cyclic monoterpene formation. However, these two monoterpene synthases yield linear instead of cyclic products. The sesquiterpene synthase (AhTPS3) is a second example of TPS-a terpene synthase harboring such motif. **Conclusions**: Our findings significantly expand our understanding of terpene biosynthesis, especially the role of RRx8W motif.

## 1. Introduction

Plants synthesize a diverse array of small organic molecules referred to as specialized metabolites (SMs) in response to changing environment [1]. Volatile organic compounds (VOCs) are one such class of SMs that are released into atmosphere or soil, serving crucial roles in communication and defense [2,3]. Once extracted from plants, VOCs are also the main constitutes of plant essential oils which find extensive application in industries such as food, agriculture, and pharmaceuticals. Structurally, VOCs could be categorized into terpenoids, phenylpropanoids, fatty acid derivatives, and amino acid derivatives, among which terpenoids are the most abundant [2].

The extremely diversified and complex structure of terpenoids is the result of catalyzation of terpene synthases (TPSs), including mono-, sesqui-, and di-terpene synthases, which converts a limited number of linear isoprenyl diphosphate substrates into complex multi-cyclic structures [4]. Phylogenetically, plant TPSs can be categorized into seven distinct subfamilies, labeled TPS-a to TPS-g [5,6]. Each of these subfamilies shares approximately 40% amino acid sequence homology among its members. The TPS-a, TPS-b, and TPS-d subfamilies predominantly include sesqui-TPSs from angiosperms, mono-TPSs from angiosperms, and mono-TPSs from gymnosperm, respectively. A distinguishing feature of the TPS-b and TPS-d subfamilies is the presence of a conserved RRx8W motif. This motif plays a crucial role in the enzyme’s function, specifically facilitating the isomerization of geranyl pyrophosphate (GPP) to (3S)-linalyl diphosphate. This isomerization is an essential step in the formation of cyclic terpenoid products [7]. Despite significant advancements in the identification and functional characterization of plant TPS genes, our understanding of these enzymes remains limited to date [8].

*Asarum heterotropoides* Fr. Schmidt var. *mandshuricum* (Maxim.) Kitag, a perennial herb belonging to the Aristolochiaceae family, is renowned for its pungent aroma and spicy flavor. Primarily found in northern China, this species also extends its presence into Korea and Japan [9]. In the traditional Chinese medicine system, it is highly valued for its efficacy in alleviating wind chill, headaches, and coughs. Although notorious aristolochic acids are ubiquitous among the plants of the Aristolochiaceae family [10], their content is relatively low in *A. heterotropoides*, particularly in its roots, which are nearly below that of detectable levels. This attribute has led to the roots of *A. heterotropoides* being recognized as one of the three medicinal plant materials based on Aristolochiaceae in the latest edition of the Chinese Pharmacopoeia [11]. The essential oil and lignans are the main active ingredients of *A. heterotropoides*, conferring antibacterial and anti-inflammatory properties [12]. While a transcriptome database of *A. heterotropoides* has recently been established [13], the biosynthetic pathways of these bioactive compounds of the species remain unexplored.

Its essential oil exhibits toxic activity against the invasive harmful species *Bactrocera dorsalis* in agriculture [14], and demonstrates a dose-dependent inhibitory effect on four strains of *Fusarium* spp. [15]. At a concentration of 2.0 mg/mL, the essential oil achieved a 100% inhibition rate against *F. avenaceum* and *F. trichothecionides* [15]. In addition, it has been observed to mitigate the severity of nasal mucosal inflammation in rats and to reduce the levels of inflammatory mediators, thereby alleviating the symptoms of allergic rhinitis [16]. The monomer components separated from the essential oil comprise a variety of chemical classes, including monoterpenes, sesquiterpenes, phenylpropanoids, and fatty acid derivatives. The monoterpene fraction is rich in β-pinene, α-phellandrene, 3-carene, and α-terpinene, whilst the sesquiterpenes include compounds such as santalene, aristolene, and β-caryophyllene [15,17].

In this investigation, we successfully cloned and functionally characterized two monoterpene synthases, AhTPS1 and AhTPS2, along with one sesquiterpene synthase, AhTPS3, from *A. heterotropoides*. All three enzymes are promiscuous TPSs that could use both GPP and farnesyl pyrophosphate (FPP) as substrates, producing monoterpenes and sesquiterpenes in vitro, respectively. By comparing the in vitro enzymatic products with the essential oil components of *A. heterotropoides*, we infer that these proteins may play a crucial role in the biosynthesis of monoterpenes and sesquiterpenes within the plant. Notably, AhTPS3 is an uncommon example of a TPS-a subfamily member that possesses the conserved RRx8W motif, which is typically associated with the TPS-b and TPS-d subfamilies.

## 2. Materials and Methods

### 2.1. Plant Materials and Chemicals

Plants of *A. heterotropoides* with the age of three years were collected from Fushun, Liaoning Province in May 2022. The plant sample was identified by Professor Guang-Wang Hu of Wuhan Botanical Garden, Chinese Academy of Sciences. GPP, FPP, and geranylgeranyl pyrophosphate ammonium salt (GGPP) were obtained from Sigma-Aldrich (St. Louis, MO, USA). Nerolidol and farnesol were purchased from Tokyo Chemical Industry (Tokyo, Japan). Linalool and geraniol were obtained from Macklin (Shanghai, China). Isopropyl β-d-thiogalactopyranoside (IPTG) was purchased from Coolaber (Beijing, China). The RNA isolation kit (DP441) was obtained from Tiangen (Beijing, China). The TransScript^®^ One-Step gDNA Removal and cDNA Synthesis SuperMix (AT311) was obtained from TransGen Biotech (Shanghai, China).

### 2.2. Essential Oil Extraction

The oil of the root of *A. heterotropoides* was extracted, based on the method for determination of essential oil in General Rule 2204 of the Chinese Pharmacopoeia [11]. Fresh root tissue (10 g) was ground and then transferred to a 1000 mL round-bottom flask connected to a distillation apparatus. About 250 mL of water was added into the flask. The essential oil was obtained by steam distillation. The crude essential oil is removed from water by using anhydrous sodium sulfate, dried three times, and stored in a brown bottle at −20 °C before analyzing by GC-MS.

### 2.3. Sequence Alignment and Phylogenetic Analysis

The Jalview(2.11.1.4) software was utilized for sequence alignment purposes. The phylogenetic tree was constructed using the maximum likelihood method. Amino acid alignments were performed with ClustalW. Bootstrap values, which indicate the statistical confidence in the tree topology, were calculated as percentages based on 1000 bootstrap replicates.

### 2.4. Molecular Docking of GPP and FPP

Models of the three-dimensional structure of AhTPS1, AhTPS2, and AhTPS3 were constructed by using Swiss-Model Server (http://swissmodel.expasy.org/ accessed on 11 April 2024), with the crystal structure of *Citrus sinensis* limonene synthase (5uv0.1.A, for AhTPS1, AhTPS2) and tobacco 5-*epi*-aristolochene synthase (5ik9.1.A, for AhTPS3) as template, which showed the highest sequence similarity with the three TPSs among the RCSB data bank proteins. The Maestro software(13.5) was employed for conducting docking simulations, utilizing GPP, FPP, and GGPP as ligands. The central coordinates for AhTPS1 were set at x = −10, y = 20, z = −15, with a binding site size of 31Å. Similarly, the central coordinates for AhTPS2 were also set at x = −10, y = 20, z = −15, but with a binding site size of 34Å. For AhTPS3, the central coordinates were x = 30, y = 70, z = 20, with a binding site size of 30Å. The results of these docking simulations were subsequently visualized using PyMOL software (2.6.0). The reliability of the model was evaluated by the GMQE scores provided by the SWISS-MODEL tool (http://swissmodel.expasy.org).

### 2.5. RNA Extraction and cDNA Synthesis

The root and leaf tissues of *A. heterotropoides* were separately pulverized in liquid nitrogen, and total RNA was isolated using an RNA isolation kit (Tiangen Biotech). Genomic DNA contamination was removed by RNase-free DNase following the manufacturer’s protocol. The RNA purity and concentration were assessed using a NanoDrop One Spectrophotometer (Thermo Fisher Scientific, Waltham, MA, USA). Subsequently, 1 μg of total RNA was used for reverse transcription with the Reverse Transcription Mix (Promega, Madison, WI, USA).

### 2.6. Prokaryotic Expression and Protein Purification

The open reading frame sequences were PCR amplified using Phanta Max Super-Fidelity DNA polymerase. The primer sequences employed for amplification are provided in Table S1. The PCR products were subsequently digested by *BamH*I and *Hind*III restriction enzymes and ligated into the *pET-32a* expression vector. Following confirmation by DNA sequencing, the recombinant plasmids were transformed into *Escherichia coli* Rosetta (DE3) for protein expression. The transformed cells were planted onto Luria–Bertani (LB) agar plates supplemented with 100 μg/mL ampicillin. A single colony was isolated and inoculated in 1 mL LB medium containing 100 μg/mL ampicillin at 37 °C and shaking at 220 rpm, until OD_600_ value reached 0.6. Protein expression was induced by the addition of 0.3 mM isopropyl β-d-1-thiogalactopyranoside (IPTG), followed by incubation at 16 °C for 12 h. The cells were collected, resuspended in 5 mL of cell lysis buffer (2.5 mM Tris-HCl, 30 mM NaCl, and 0.2 mM imidazole, pH 7.0), and disrupted using a constant cell disruption system at a pressure of 19.0 Kpsi. After removal of residue by beingcentrifuged at 12,000 rpm for 15 min, the recombinant proteins were purified using Ni-NTA resin (Thermo Fisher Scientific), according to the manufacturer’s instructions. Protein concentrations were quantified using the Bradford assay, with bovine serum albumin (BSA) as the standard.

### 2.7. Enzyme Assay

The TPS activity was assayed following a previously reported method with slight modifications [18]. The activity of the recombinant proteins was evaluated in a 500 μL reaction volume containing a buffer with 50 mM HEPES (pH 7.5), 5 mM MgCl_2_, 4 mM dithiothreitol, 40 μM GPP or FPP or GGPP, and 100 μg of protein. The corresponding proteins boiled in 100 °C water for 10 min were used as negative controls. The reactions were incubated at 30 °C for 3 h. Subsequently, the reaction mixtures were extracted with 500 μL of hexane, then it is dried by using anhydrous sodium sulfate three times, and the products were analyzed by using gas chromatography–mass spectrometry (GC–MS).

### 2.8. Gas Chromatography–Mass Spectrometry Analysis

The *A. heterotropoides* essential oil and enzymatic products were analyzed on a SHIMADZU QP2020NX Series GC-MS, with the carrier gas helium at 1 mL/min, injection volume of 1 μL, and separated on a SHIMADZU-Rtx-5MS column (length 30.0 m, diameter: 250.00 μm, film thickness: 0.25 μm). For *A. heterotropoides* essential oil analysis, a temperature program was used as follows: initial temperature of 60 °C (3 min hold), increased to 75 °C at 1 °C/min, then raised to 90 °C at a rate of 0.5 °C/min, followed by ramping to 180 °C at a rate of 1 °C/min, and finally elevated to 220 °C and held for 10 min. For enzymatic assay, the following temperature program was used: initial temperature of 60 °C (3 min hold), increased to 180 °C by 4 °C/min, and ramp to 220 °C by 2 °C/min (10 min hold). Compounds were identified by comparing with available standards, and calculated retention indices with those of reference.

### 2.9. Subcellular Localization Analysis

The full-length cDNAs of *AhTPS1*, *AhTPS2*, and *AhTPS3* were ligated into the PBI121-EGFP vector. The resultant plasmid was transformed into GV3101 (pSoup-p19), and the clones harboring recombinant plasmids were cultured in 10 mL LB liquid medium containing 25 mg/mL rifampicin, and 50 mg/mL kanamycin at 28 °C, and shaking at 220 rpm for 36 h. After centrifugation at 7000× *g* for 2 min, cells were gently resuspended in MES buffer containing 150 mM acetosyringone to make a solution with a final OD_600_ of 1.2 for each transformant. After incubation at room temperature for about 2 h, the prepared solution was aspirated into a sterile needle-free syringe (1 mL) to infiltrate the leaves of 5-week-old *N. benthamiana*. Plants infiltrated with solutions of GV3101 harboring empty PBI121-EGFP (for *AhTPS1*, *AhTPS2*) and empty PBI121-EGFP/pCAMBIA1300-35S-mCherry (for *AhTPS3*) were used as negative controls. A total of 36~48h after infiltration, the transgenic tobacco leaves were observed and imaged under a confocal laser scanning microscope (Olympus, Tokyo, Japan).

## 3. Results

### 3.1. Essential Oil of A. heterotropoides Root Determined by GC-MS

**Figure 1:**
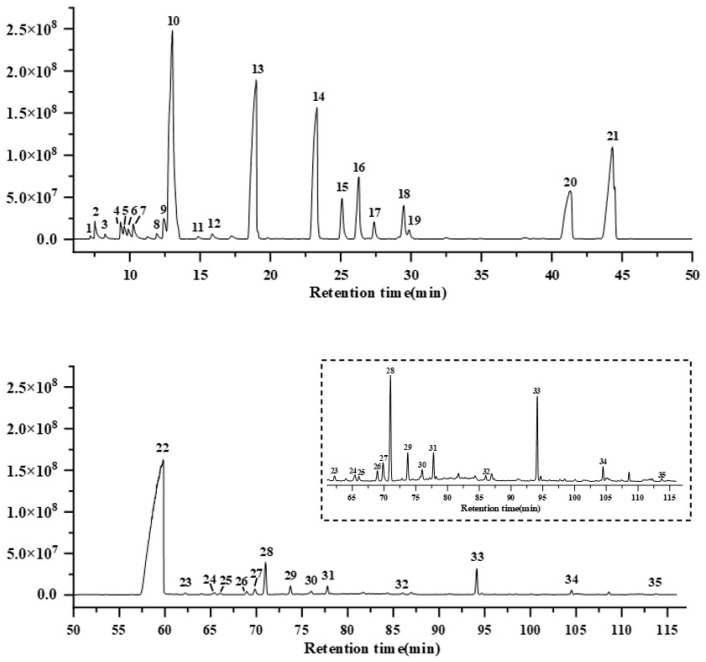
**The components of essential oil of *A. heterotropoides* root analyzed by GC-MS.** Peaks are 1, *α*-phellandrene; 2, α-pinene; 3, camphene; 4, *β*-phellandrene; 5, *β*-pinene; 6, linalool; 7, *β*-myrcene; 8, (+)-4-carene; 9 *, p-cymene; 10, eucalyptol; 11, *γ*-terpinene; 12, linalool oxide; 13, isopulegol; 14, 1-isopropenyl-4-methyl-1,2-cyclohexanediol; 15, isobornyl acetate; 16, isoborneol; 17, terpinen-4-ol; 18, *α*-terpineol; 19, estragole; 20 *, Cinnamaldehyde; 21 *, safrole; 22 *, eugenol; 23, copaene; 24, *β*-elemene; 25, vanillin; 26 *, methyleugenol; 27, caryophyllene; 28, *α*-santalol; 29, *α*-bergamotene; 30, humulene; 31, *β*-santalol; 32, levomenol; 33, nerolidol; 34, guaiol; 35 *, coniferyl aldehyde. Numbers marked with * represent aromatic compounds.

The essential oil isolated from root of *A. heterotropoides* is its main active ingredient. To analyze the components of the oil, GC-MS analysis on the extracted essential oil was performed, which led to detection of 28 terpenoids and 7 phenylpropanoids (Figure 1 and Figure S2), most of which were reported in previous results [19,20,21]. Among aromatic compounds, eugenol, safrole ether, and cinnamaldehyde were the main components, while among terpenoids, eucalyptol, isopulegol, isoborneol, α-santalol, α-terpineol, and nerolidol were the most abundant components.

### 3.2. Cloning of Monoterpene and Sesquiterpene Synthases

**Figure 2:**
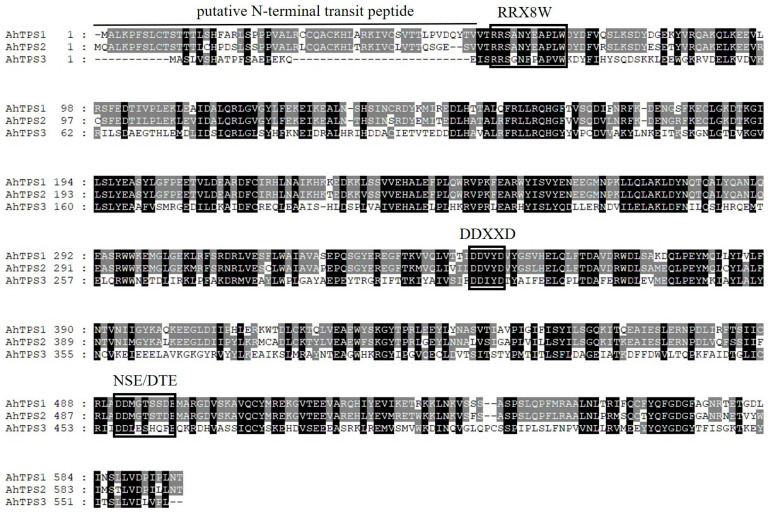
**Alignment of deduced amino acid sequences of AhTPS1, AhTPS2, and AhTPS3.** The conserved RRx8W, DDXXD, and NSE/DTE domains are framed. The sequences were aligned by Clustal W.

From a cDNA library of *A. heterotropoides,* three cDNAs, namely *AhTPS1*, *AhTPS2*, and *AhTPS3*, (GenBank accession numbers are PQ732185, PQ732186, and PQ732187, respectively) were successfully isolated. They encode proteins consisting of 618, 617, and 583 amino acids, respectively, all of which harbor the conserved DDXXD and NSE/DTE ion-binding domain (Figure 2). All three proteins possess a conserved RRx8W domain, which is characteristic of plant monoterpene synthases [22,23]. There are 53, 52, and 21 residues upstream of the of *AhTPS1*, *AhTPS2*, and *AhTPS3*, respectively, which suggested that the putative plastid-targeting signal sequence found at the N-terminus of typical plant monoterpene synthases is present in AhTPS1 and AhTPS2 but is notably absent in AhTPS3, because the signal peptide of monoterpene synthases usually contains 50–70 amino acids [5].

**Figure 3:**
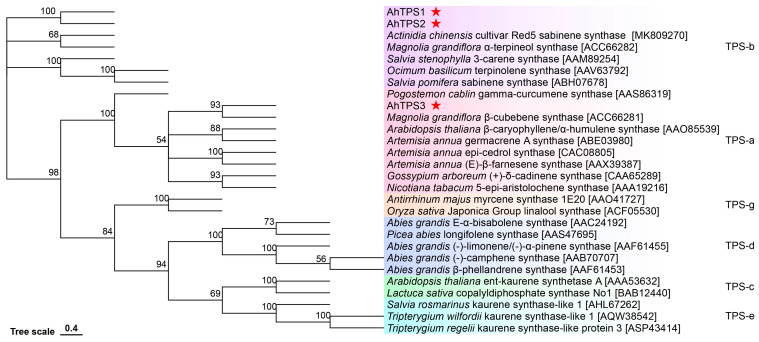
**Phylogenetic analysis of three TPSs from *A. heterotropoides* with other plant terpene synthases of different clades**.

Phylogenetic analysis of these synthases (Figure 3) revealed that AhTPS1 and AhTPS2 are classified within the TPS-b subfamily and are clustered with other monoterpene synthases, such as (−)-β-pinene synthase (*Artemisia annua*), α-terpineol synthase (*Magnolia grandiflora*), sabinene synthase (*Salvia pomifera*), and E-β-ocimene synthase (*Lotus japonicus*). In contrast, AhTPS3 was positioned within the TPS-a subfamily and grouped alongside sesquiterpene synthases, including β-cubebene synthase (*Magnolia grandiflora*), (E)-β-farnesene synthase (*A. annua*), β-caryophyllene/α-humullene synthase (*A. annua*), and epi-cedrol synthase (*A. annua*). These findings suggest that AhTPS1 and AhTPS2 are likely monoterpene synthases, while AhTPS3 functions as a sesquiterpene synthase. However, the phylogenetic placement of AhTPS3 hints at its potential evolutionary origin from a monoterpene synthase ancestor as it harboring a RRx8W motif.

### 3.3. Molecular Docking

**Figure 4:**
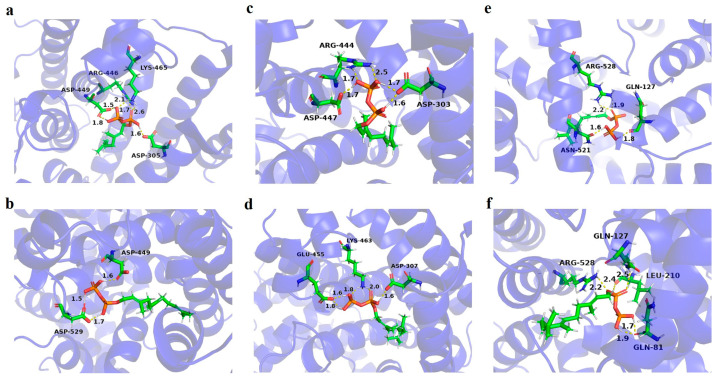
**Molecular modeling of AhTPS1, AhTPS2, and AhTPS3, docked with GPP and FPP.** (**a**) AhTPS1 bound with GPP. (**b**) AhTPS1 bound with FPP. (**c**) AhTPS2 bound with GPP. (**d**) AhTPS2 bound with FPP. (**e**) AhTPS3 bound with GPP. (**f**) AhTPS3 bound with FPP.

Some TPSs exhibit catalytic promiscuity, capable of converting isoprenyl diphosphate substrates of varying lengths into final products. To investigate whether the isolated TPSs from *A. heterotropoides* are also promiscuous enzymes, we employed molecular docking to assess the binding affinity of GPP, FPP, and GGPP to the TPSs in question. Consistent with the phylogenetic analysis, all three TPSs tested did not accommodate GGPP as a ligand. However, they all demonstrated the potential to bind both GPP and FPP, indicating that they might be promiscuous TPSs with both monoterpenes and sesquiterpenes synthases activity (Figure 4).

As depicted in Figure 2, the residues Asp305, Arg446, Asp449, and Lys465 of AhTPS1 were found to interact with the phosphate group of GPP, resulting in a binding energy of −6.952 kcal/mol. Meanwhile, Asp449 and Asp529 showed interaction with the phosphate group of FPP, with a binding energy of −7.987 kcal/mol. For AhTPS2, the residues Asp303, Arg444, and Asp447 were involved in binding to the phosphate group of GPP, with a binding energy of −6.816 kcal/mol. Additionally, Asp307, Glu455, and Lys463 interacted with the phosphate group of GPP, exhibiting a binding energy of −6.310 kcal/mol. In the case of AhTPS3, the residues Gln127, Asn521, and Arg528 coordinated with the phosphate group of GPP, with a binding energy of −4.023 kcal/mol. However, Gln127, Arg528, Leu210, and Gln81 interacted with the phosphate group of FPP, with a binding energy of −2.691 kcal/mol.

These findings suggest that AhTPS1, AhTPS2, and AhTPS3 may be catalytically promiscuous enzymes, with the ability to bind and potentially convert both GPP and FPP into various products.

To validate the reliability of the docking models, the GMQE scores of three models were calculated, which is expressed as a number between 0 and 1 and higher members indicate higher reliability of the model [24]. The models of AhTPS1, AhTPS2, and AhTPS3 showed scores of 0.75, 0.76, and 0.76, respectively, comparable to the previous reported PamTps1 model of 0.82 [25], suggesting the reliability of these three models.

### 3.4. Enzymatic Activities of AhTPS1, AhTPS2, and AhTPS3

**Figure 5:**
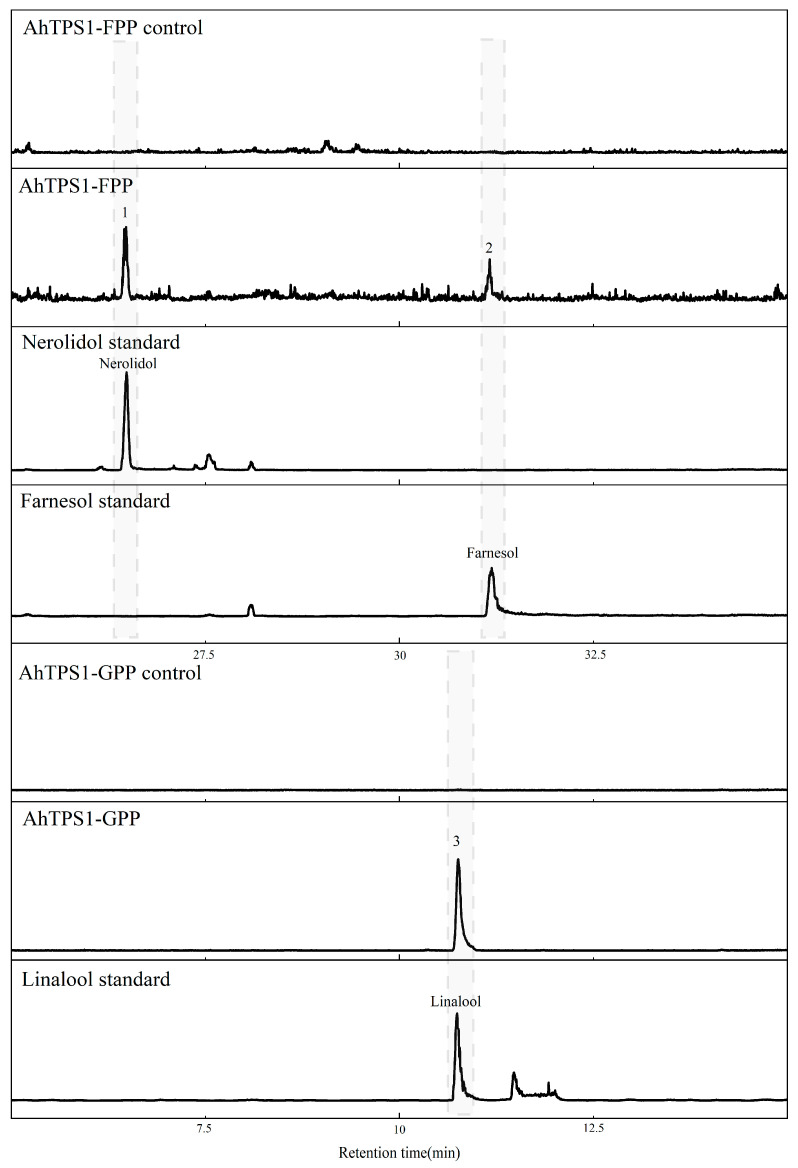
**Identification of the products catalyzed by recombinant AhTPS1 in vitro.** Total ion chromatograms from GC-MS analysis of hexane extracts of AhTPS1 after incubation with GPP and FPP as substrate.

**Figure 6:**
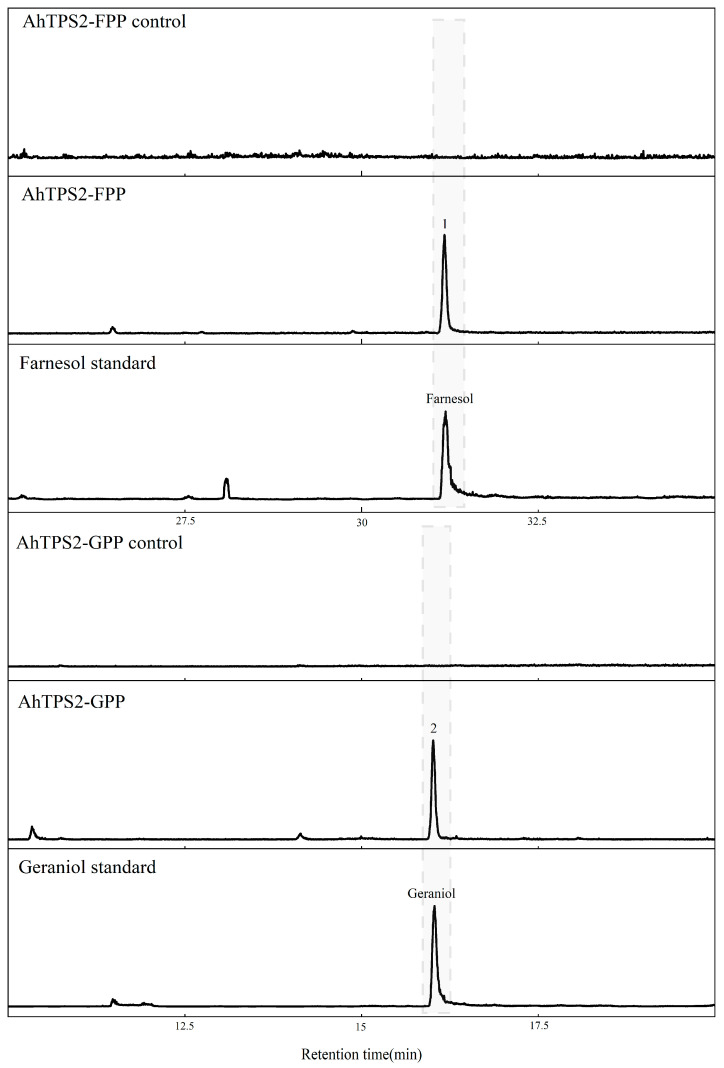
**Identification of the products catalyzed by recombinant AhTPS2 in vitro.** Total ion chromatograms from GC-MS analysis of hexane extracts of AhTPS2 after incubation with GPP and FPP as substrate.

**Figure 7:**
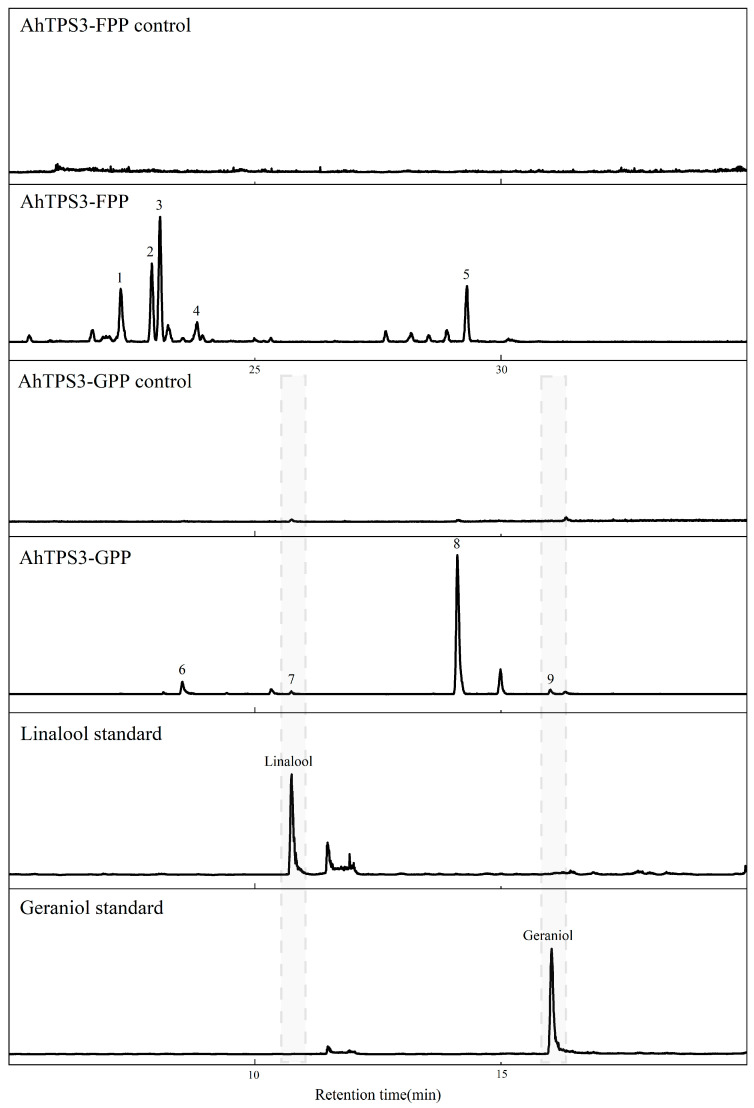
**Identification of the products catalyzed by recombinant AhTPS3 in vitro.** Total ion chromatograms from GC-MS analysis of hexane extracts of AhTPS3 after incubation with GPP and FPP as substrate.

To elucidate their function and verify the docking results, the purified proteins of AhTPS1, AhTPS2, and AhTPS3 were incubated with GPP, FPP, and GGPP to test their activity on these substrates. The products were identified by comparing with available standards, and those without standard were determined by comparing calculated retention indices with those of reference. As a result, none of these TPSs were active towards GGPP in our enzymatic assay. When FPP was added, AhTPS1 converted it into two products, nerolidol and farnesol (Figure 5); meanwhile, AhTPS2 yielded a single sesquiterpene product, farnesol (Figure 6), and AhTPS3 generated five sesquiterpenes such as patchoulene, maaliene, blunesene, acorenol, and an unknown sesquiterpene corresponding to peaks 2, 3, 4, 5, and 1 in Figure 7 (Figure 5, Figure 6 and Figure 7 and Figure S2, and Table S2). The structure of the unknown sesquiterpene may be related to that of himachala-2,4-diene, as these two compounds showed similar MS spectrums, but with different retention indices. Meanwhile, when GPP was used as a substrate, AhTPS1 and AhTPS2 could catalyze the formation of single monoterpene products, linalool and geraniol, respectively, while AhTPS3 produced four monoterpenes including limonene, linalool, an unknown monoterpene, and geraniol corresponding to peaks 6, 7, 8, and 9 in Figure 7 (Figure 5, Figure 6 and Figure 7 and Figure S2, and Table S2). Due to the similar MS spectrums and different retention indices, the structure of the unknown monoterpene may be analogous to that of ocimenol. The above data confirmed the docking results of the promiscuous TPSs of AhTPS1, AhTPS2, and AhTPS3, that they could use both GPP and FPP as substrates in in vitro enzymatic assay.

### 3.5. Subcellular Localization of AhTPS1, AhTPS2, and AhTPS3

**Figure 8:**
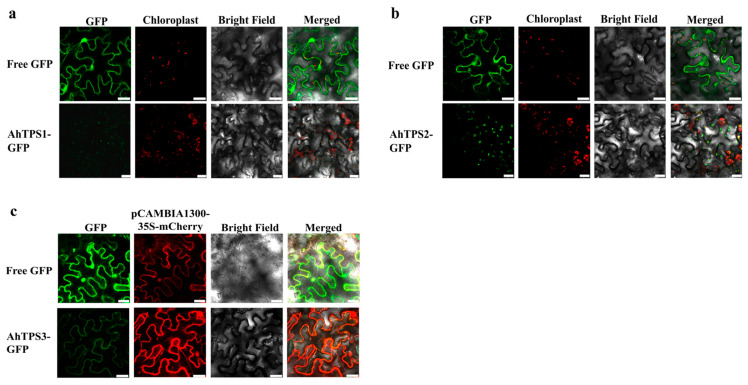
**Subcellular localization of AhTPS1, AhTPS2, and AhTPS3.** Full-length AhTS1 (**a**), AhTPS2 (**b**), and AhTPS3 (**c**) fused with GFP were transiently expressed in *N. benthamiana* leaves and visualized by laser confocal microscopy. The signal of GFP is shown in green, the autofluorescence of chloroplast and Pcambia1300-35S-mCheery are shown in red, scale bar: 25 μm.

Although AhTPS1, AhTPS2, and AhTPS3 were predicted to be chloroplasts-, chloroplasts-, and cytosol-localized proteins, respectively, by comparing their signal peptide lengths, it was important to verify their subcellular localization experimentally to clarify the above-mentioned multiple biochemical functions. Therefore, the full-length cDNAs of these three genes were ligated into the PBI121-EGFP vector, then transiently expressed in *N*. *benthamiana*. Confocal laser scanning microscopy analysis revealed that the green fluorescence signals of AhTPS1-GFP and AhTPS2-GFP merge well with chloroplast auto-fluorescence (Figure 8a,b). These results indicated that AhTPS1 and AhTPS2 were localized to the chloroplast. However, the green fluorescence signal of AhTPS3-GFP colocalized with the red fluorescence signal of pCAMBIA1300-35S-mCherry (Figure 8c), suggesting a cytosol localization of AhTPS3. Therefore, the above data indicated that AhTPS1 and AhTPS2 were chloroplast-targeted monoterpene synthases, and AhTPS3 was cytosol-localized sesquiterpene synthase.

## 4. Discussion

The *A. heterotropoides* plant boasts a rich composition, with essential oils and lignans standing out as the primary bioactive constitutes. Within the plant kingdom, these compounds serve as vital tools for biological defense, warding off herbivores and pathogens. Their pharmacological properties, particularly anti-inflammatory and analgesic effects, have garnered substantial research interest. While extensive studies have been conducted on the chemical profiling of this species [26,27,28], our understanding of the biosynthetic pathways leading to these medicinally valuable compounds remains limited [29,30].

In the present study, we have successfully cloned and biochemically characterized terpene synthases (TPSs) from *A. heterotropoides* for the first time. This achievement not only broadens our understanding of terpene biosynthesis but also paves the way for in-depth exploration into the biosynthesis of bioactive terpenoids within this species. Notably, approximately one-third of plant monoterpene synthases are known to convert GPP into acyclic products [31]. AhTPS1 and AhTPS2 belong to this group, as they specifically produce linear linalool and geraniol, respectively, as their sole products. The detection of linalool in the essential oil extracted from *A. heterotropoides* corroborates the physiological role of AhTPS1. The presence of linalool is rather widespread as it is found in a number of plants belonging to families Lamiaceae (mints, scented herbs), Lauraceae (laurels, cinnamon, rosewood), and Rutaceae (citrus fruits). Linalool has been reported for insecticidal activity against stored product insects and has potential against cat fleas [32]. The detection of linalool in *A. heterotropoides* and other plants suggested that it may play an important role in defense.

While AhTPS1 demonstrated the capacity to generate nerolidol when FPP was supplied in vitro—a compound also found in *A. heterotropoides* essential oil—it is improbable that this enzyme contributes to its *in planta* synthesis. This conclusion stems from AhTPS1’s localization to the chloroplast, and the cytosolic prevalence of FPP. The functional correlation between in vitro activity and in vivo function becomes more intricate with AhTPS3. In vitro, it produces linalool, suggesting a potential *in planta* role, especially considering the possible accessibility of cytosolic GPP through transport from chloroplasts or plasmodesmata [33,34], or synthesis by bifunctional geranyl/farnesyl diphosphate synthases in the cytosol [35]. Concurrently, AhTPS3 may be responsible for the production of patchoulene, blunesene, and limonene, all detected as in vitro products and reported constitutes of *A. heterotropoides* essential oil [26,27,28], despite their absence in our oil extractions, as a result of low expression level of *AhTPS3* presumably. It is well known that the biosynthesis of plant secondary metabolites is rather dynamic, greatly influenced by their environmental and developmental stage [36,37]. Notably, the terpene profiles of *A. heterotropoides* essential oil are different among ours and those of the reported, which may be a result of different TPSs expression level *in plantain planta* that depend on various environmental and developmental stages.

It is of interest that AhTPS1 and AhTPS2 yield only one product with GPP as substrate, whilst AhTPS3 generates five sesquiterpenes. From a mechanistic point of view, a TPS provides a template for a cyclization cascade [4]. Thus, the active pocket of a specific TPS is relatively small and could precisely control the reaction cascade, leading to the generation of one product. On the other hand, the active site volume of a promiscuous TPS is much larger, accommodated multiple substrates or intermediate conformations, resulting in the formation of multiple products [38]. Indeed, AhTPS3 has the largest catalytic pocket (820.56 Å^3^) than those of AhTPS1 (603.32 Å^3^) and AhTPS2 (624.87 Å^3^).

All TPSs containing the RRx8W motif belong to the TPS-b and TPS-d terpene clades, with the exception of one synthase, (*E*,*E*)-α-farnesene synthase from *Cucumis sativus*, which is part of the TPS-a clade [39,40]. AhTPS3, a member of the TPS-a subfamily, also contains this domain, providing another example of these rule-breaking TPSs. Unlike (*E*,*E*)-α-farnesene synthase that produces linear sesquiterpene α-farnesene and monoterpene (*E*)-β-ocimene [39], AhTPS3 yields five cyclic sesquiterpenes and one cyclic monoterpene in addition to three linear monoterpenes. It is likely that AhTPS3 was derived directly from monoterpene synthases by losing their signal peptide and broadening substrate acceptance to include FPP. The lack of RRx8W motif has been regarded as a feature of multi-substrate TPS previously [40]; however, all of the three TPSs reported here contain this motif but could use both GPP and FPP as substrates, also deviated from this rule. Interestingly, although AhTPS1 and AhTPS2 contain the RRx8W motif crucial for cyclic terpene production, they only produce acyclic monoterpenes and sesquiterpenes when GPP and FPP are used as substrates. The above observation provides exception for cyclic and mono-substrate role of RRx8W, suggesting significant modifications in the catalytic mechanisms of the three enzymes.

## Data Availability

The original contributions presented in the study are included in the article/Supplementary Material. Further inquiries can be directed to the corresponding authors.

## Supplementary Materials

The following supporting information can be downloaded at: https://www.mdpi.com/article/10.3390/metabo15110753/s1, Figure: S1. SDS-PAGE gels of AhTPS1, AhTPS2, and AhTPS3 proteins, Figure S2. MS spectrum of compounds identified in this study, Table S1. Primers used in this investigation, Table S2. Retention indices of products generated by the recombinant enzymes.
