# Supplementary material for: Isolation and Characterization of Two Monoterpene Synthases and a Sesquiterpene Synthase from Asarum heterotropoides
Source: Metabolites. 2025 Nov 20;15(11):753. doi: 10.3390/metabo15110753 (PMC12654742; doi:10.3390/metabo15110753)
Supplement: Supplementary file 1 [file metabolites-15-00753-s001.zip › metabolites-3882377-supplementary.pdf]

Supplementary Materials

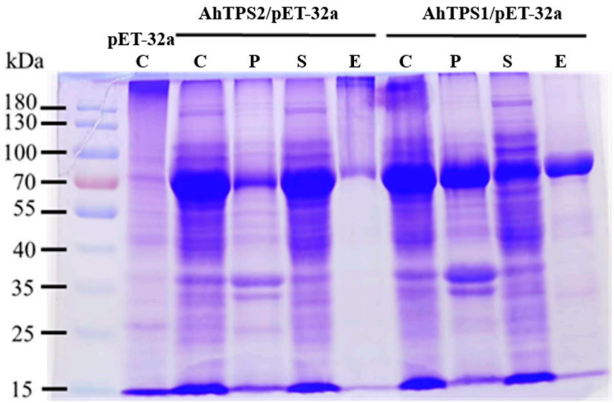

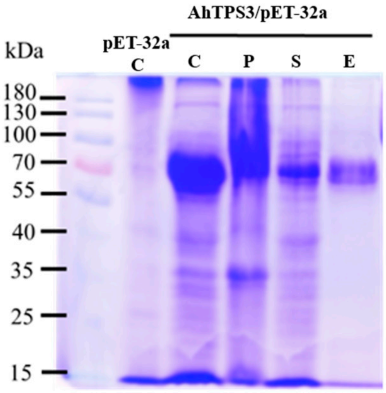

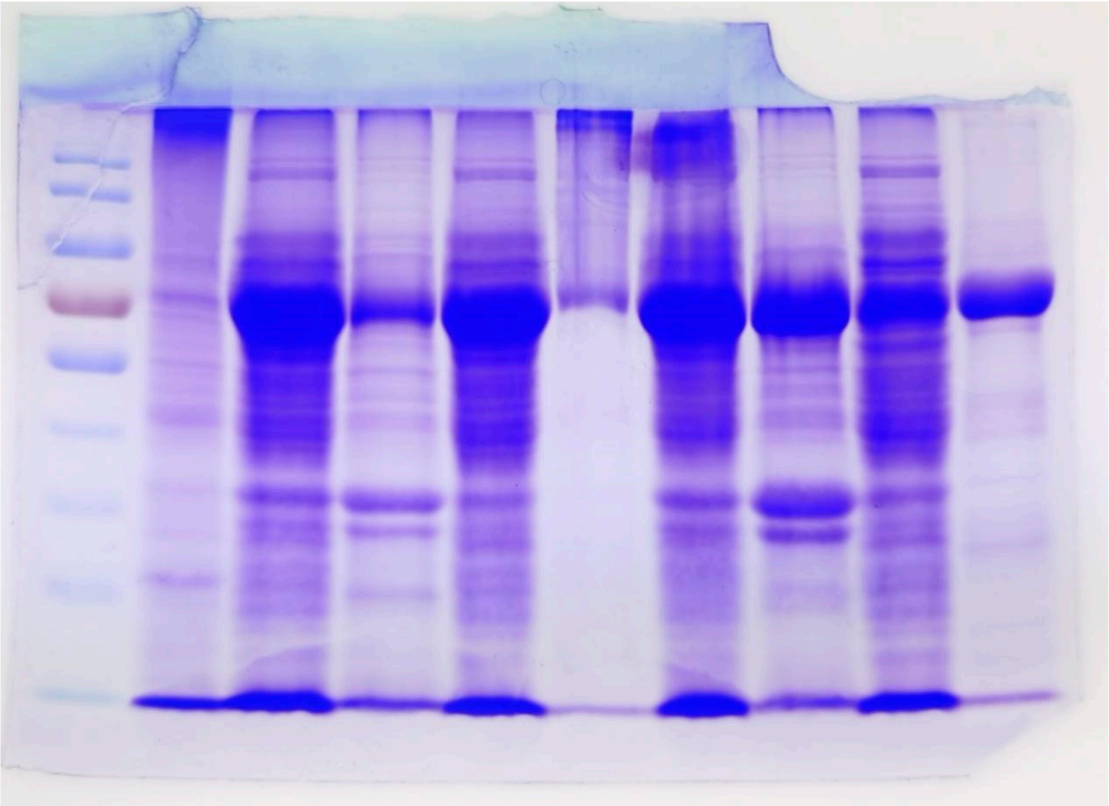

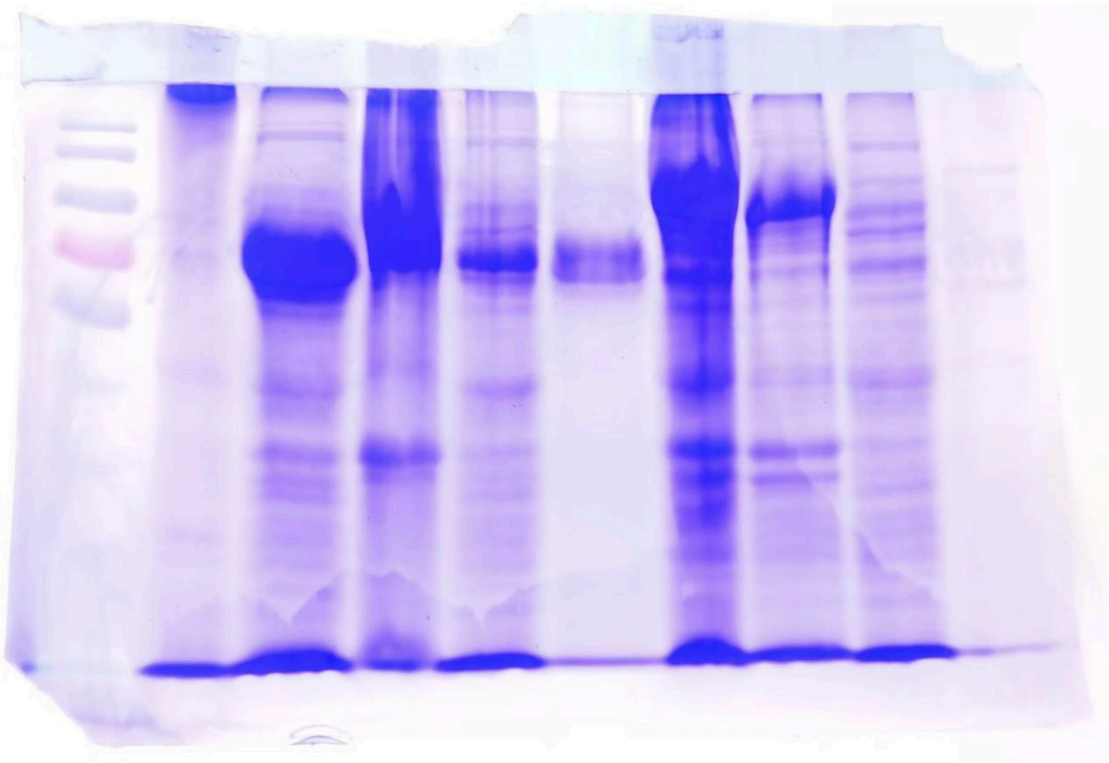

Figure S1. SDS-PAGE gels of AhTPS1, AhTPS2, and AhTPS3 proteins. The prokaryotically expressed proteins were purified and subjected to SDS-PAGE gel electrophoresis. The crude (C), supernatant (S), pellet (P), eluate (E) proteins were showed. The intact gels were shown below.

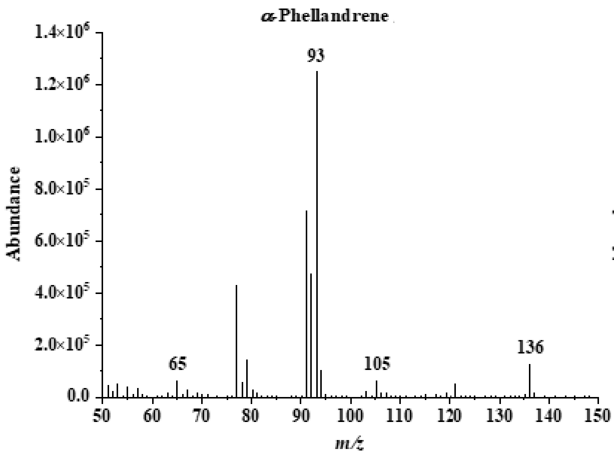
α-Phellandrene
93
65
105
136
Abundance
m/z

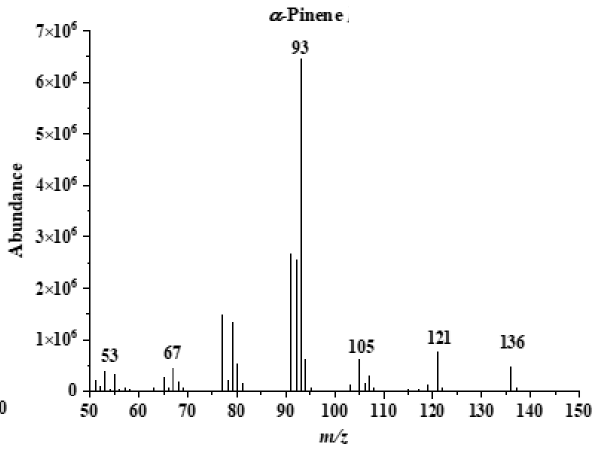
α-Pinene
93
53
67
105
121
136
Abundance
m/z

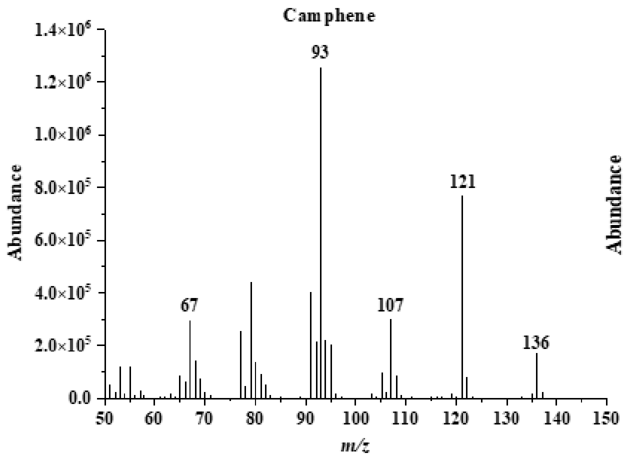
Camphene
93
121
67
107
136
Abundance
m/z

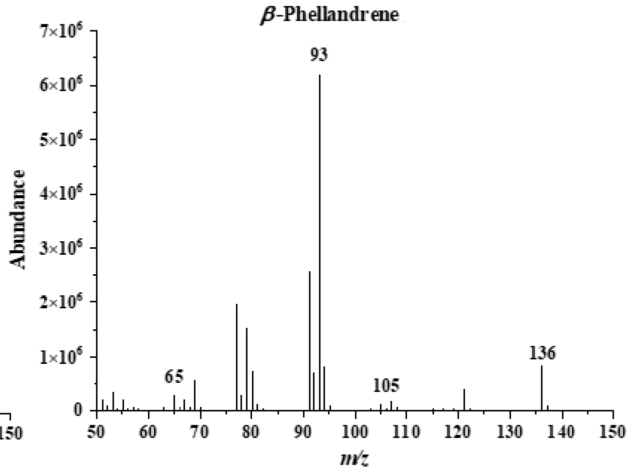
β-Phellandrene
93
65
105
136
Abundance
m/z

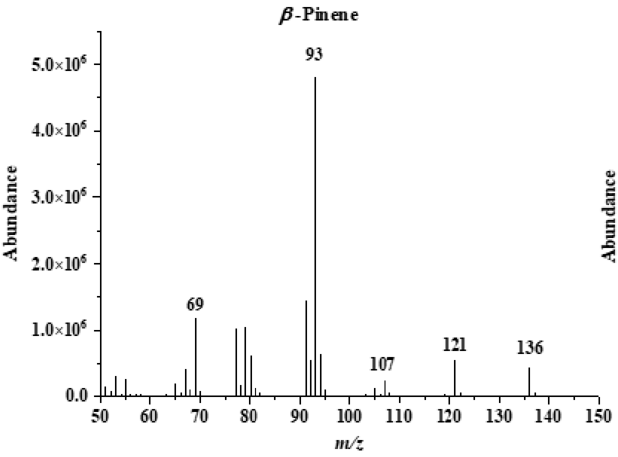
β-Pinene
93
69
107
121
136
Abundance
m/z

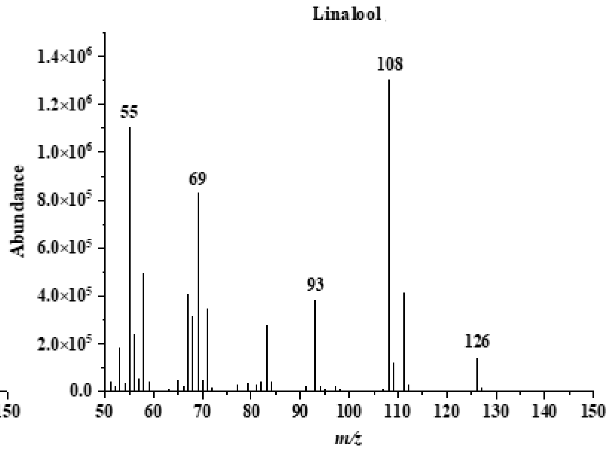
Linalool
108
55
69
93
126
Abundance
m/z

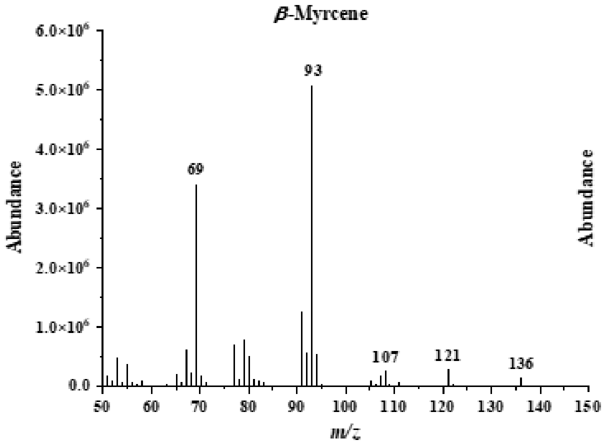
β-Myrcene
Abundance
m/z
69
93
107
121
136

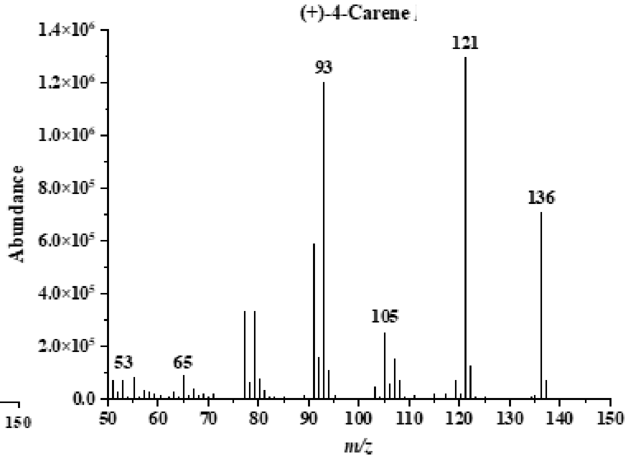
(+)-4-Carene
Abundance
m/z
53
65
93
105
121
136

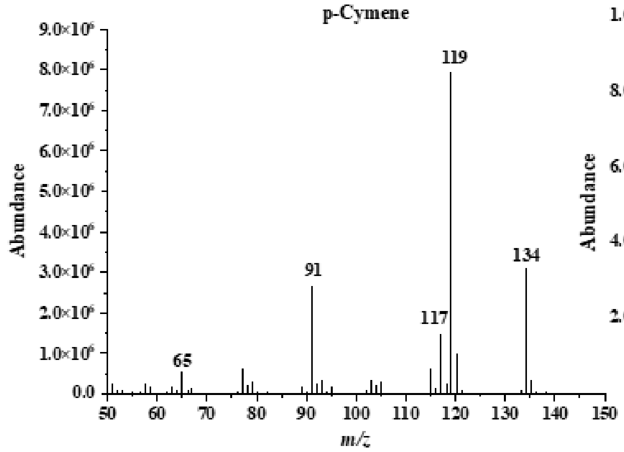
p-Cymene
Abundance
m/z
65
91
117
119
134

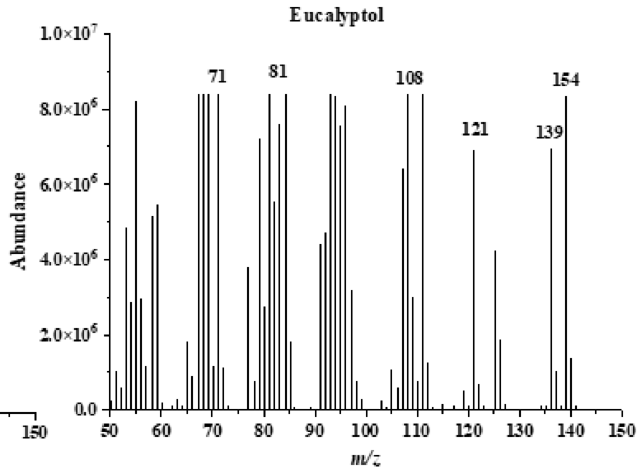
Eucalyptol
Abundance
m/z
71
81
108
121
139
154

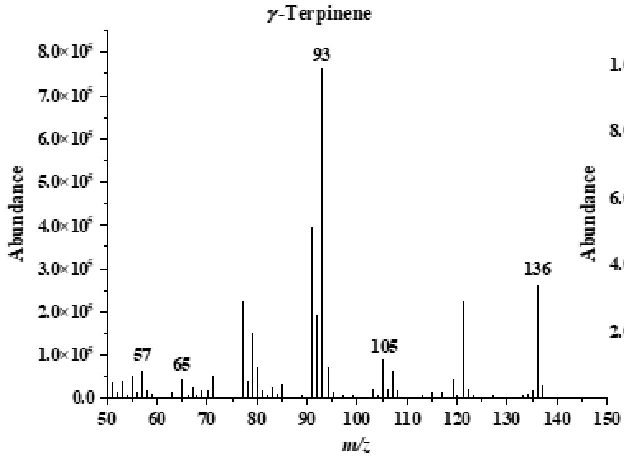
γ-Terpinene
Abundance
m/z
57
65
93
105
136

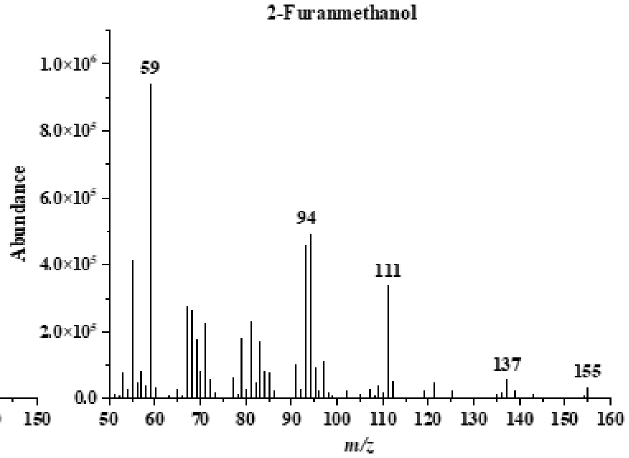
2-Furanmethanol
Abundance
m/z
59
94
111
137
155

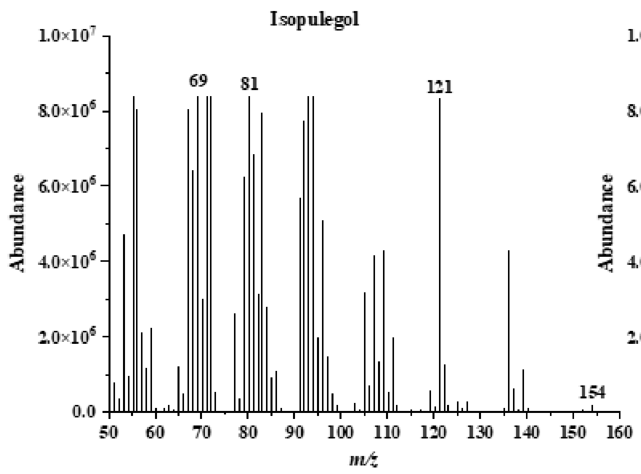
Isopulegol
Abundance
m/z
69
81
121
154

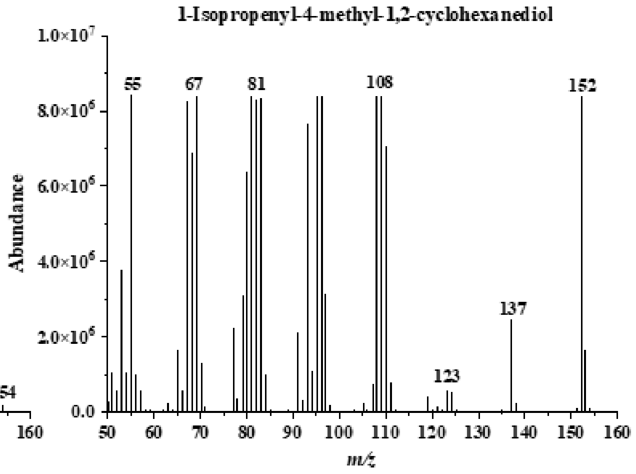
1-Isopropenyl-4-methyl-1,2-cyclohexanediol
Abundance
m/z
55
67
81
108
152
137
123

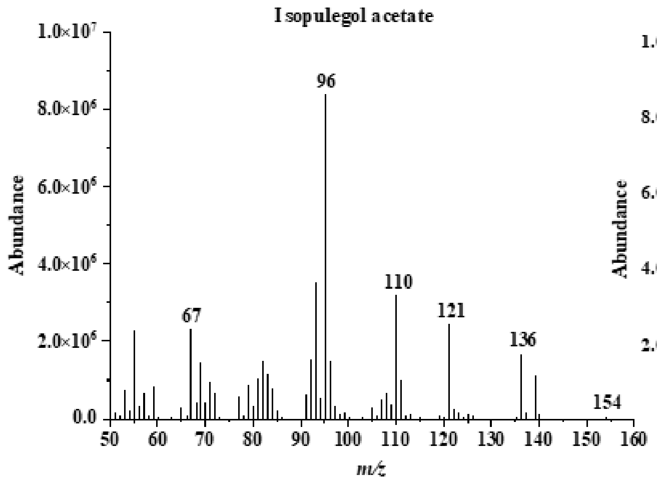
Isopulegol acetate
Abundance
m/z
96
110
121
136
67
154

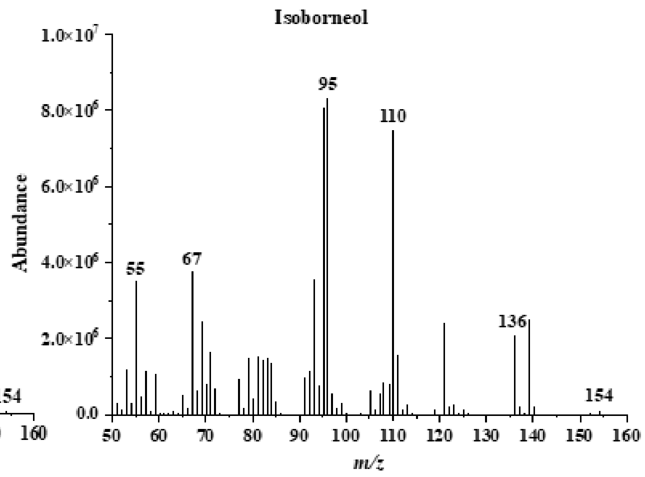
Isoborneol
Abundance
m/z
95
110
55
67
136
154

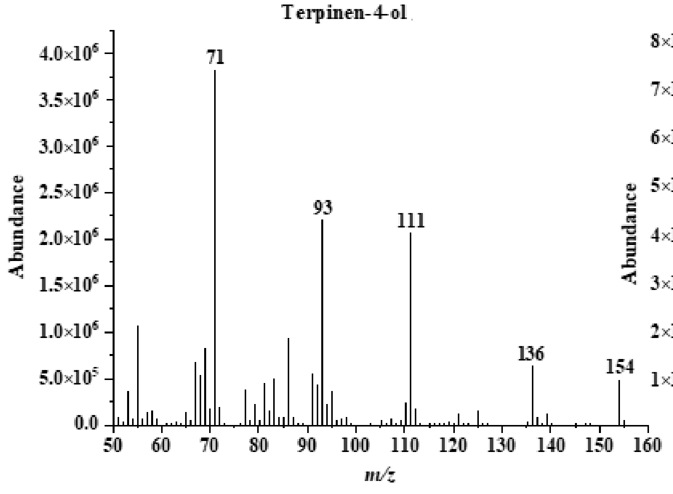
Terpinen-4-ol
Abundance
m/z
71
93
111
136
154

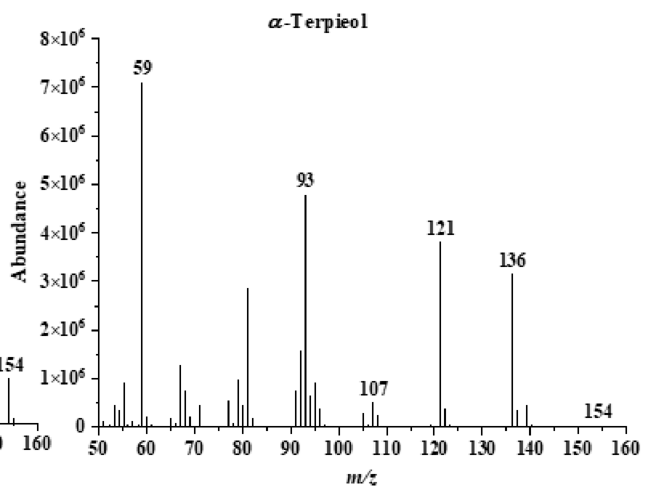
α-Terpieol
Abundance
m/z
59
93
121
136
107
154

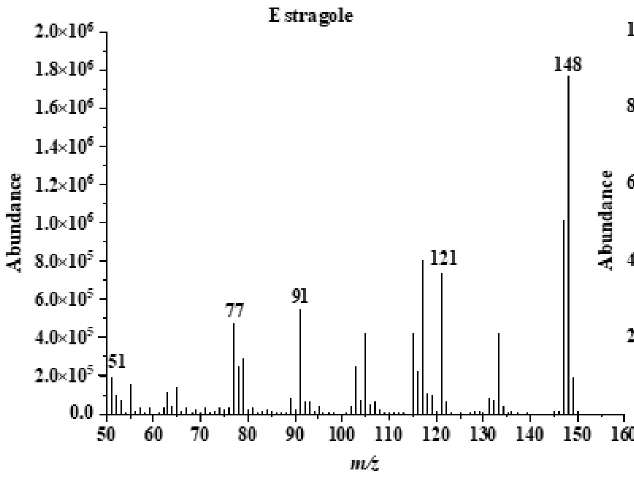
Estragole
Abundance
m/z
51
77
91
121
148

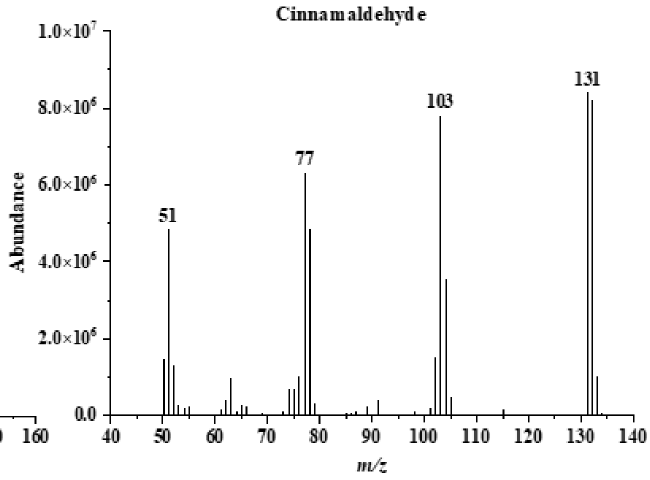
Cinnamaldehyde
Abundance
m/z
51
77
103
131

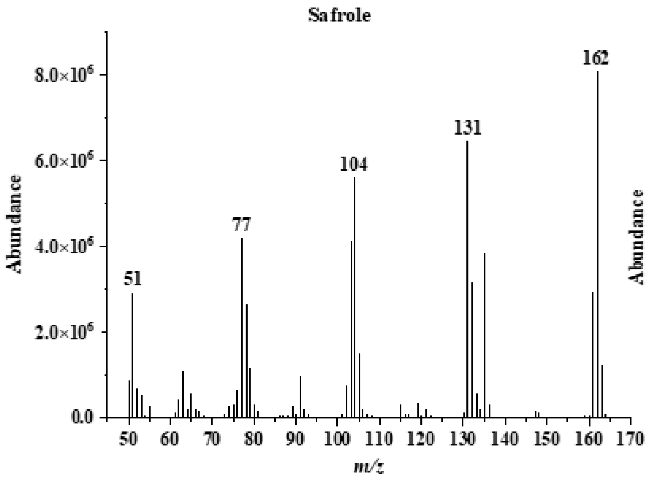
Safrole
Abundance
m/z
51
77
104
131
162

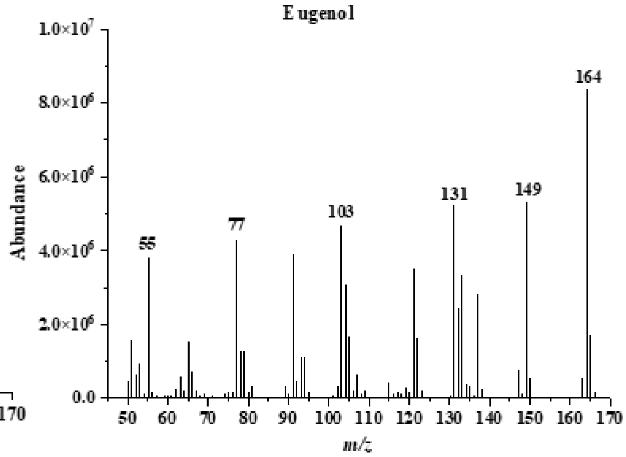
Eugenol
Abundance
m/z
55
77
103
131
149
164

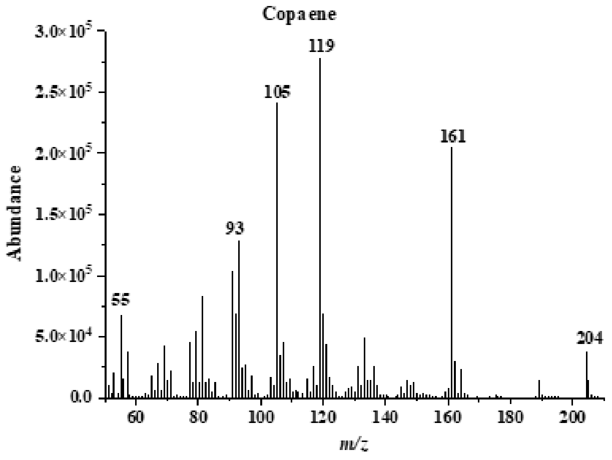
Copaene
Abundance
m/z
55
93
105
119
161
204

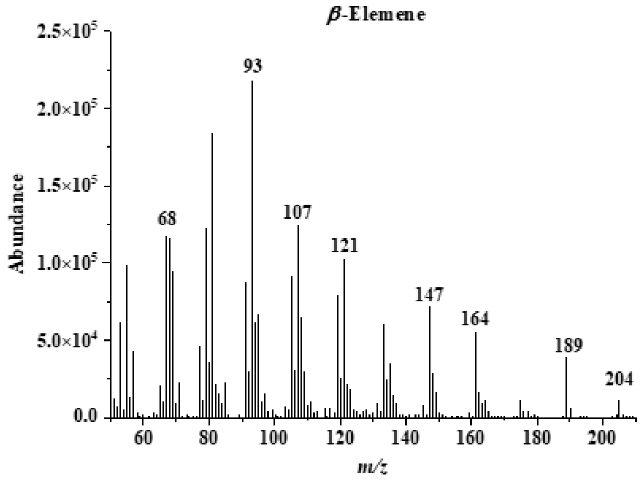
β-Elemene
Abundance
m/z
68
93
107
121
147
164
189
204

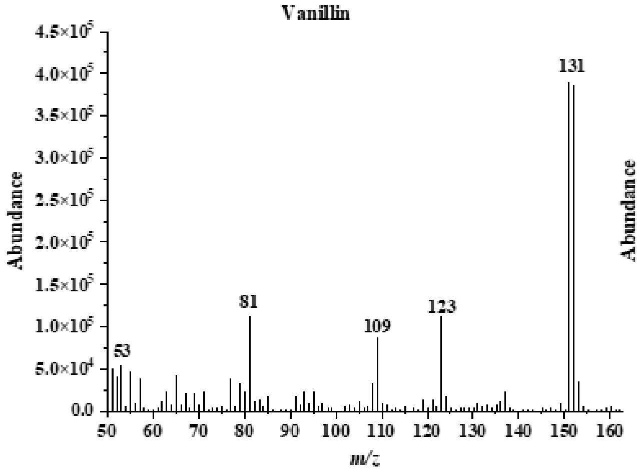
Vanillin
Abundance
m/z
53
81
109
123
131

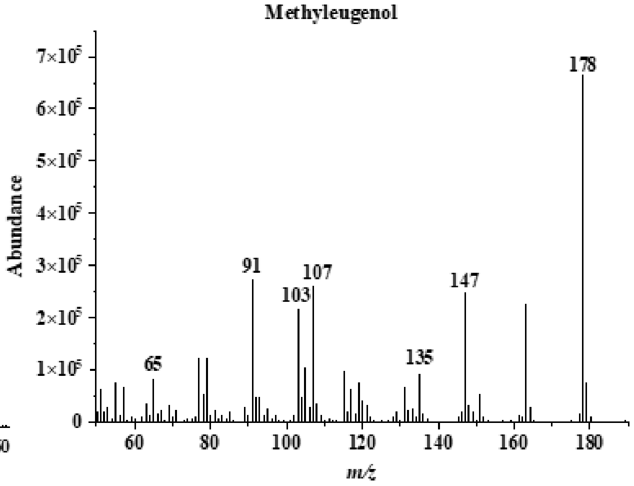
Methyleugenol
Abundance
m/z
65
91
103
107
135
147
178

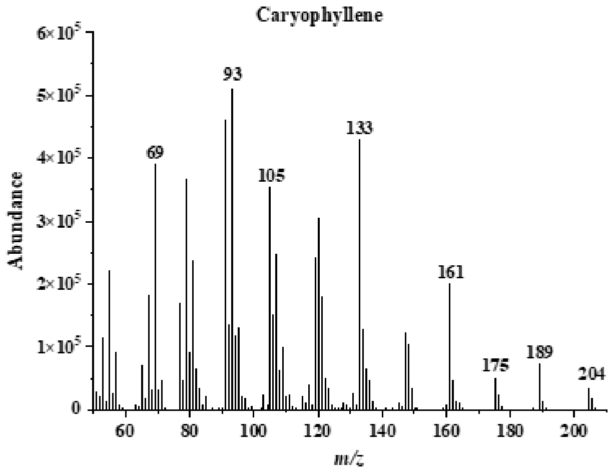
Caryophyllene
Abundance
m/z
69
93
105
133
161
175
189
204

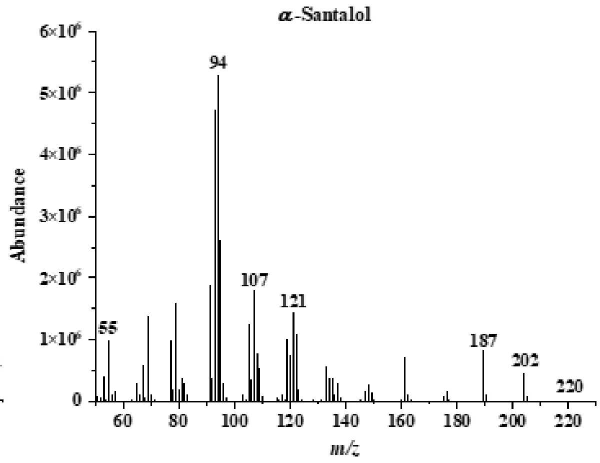
α-Santalol
Abundance
m/z
55
94
107
121
187
202
220

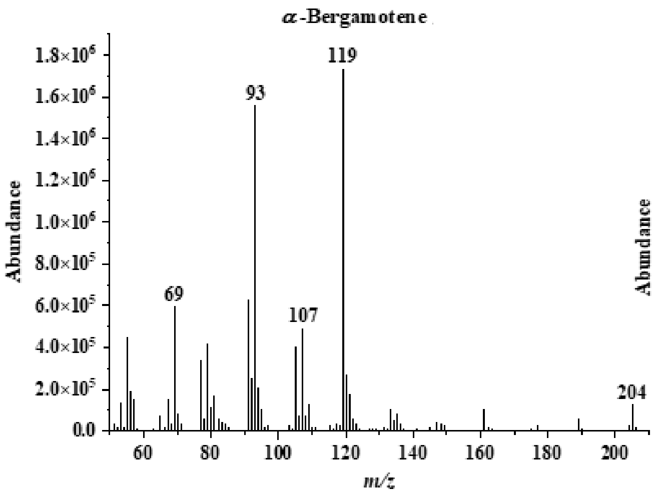
α-Bergamotene
Abundance
m/z
69
93
107
119
204

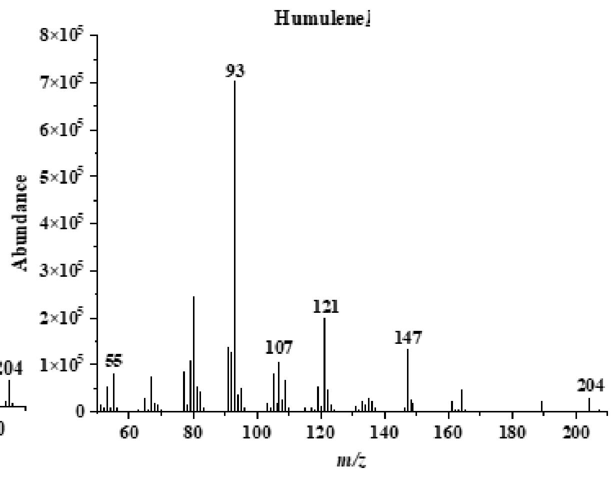
Humulene
Abundance
m/z
55
93
107
121
147
204

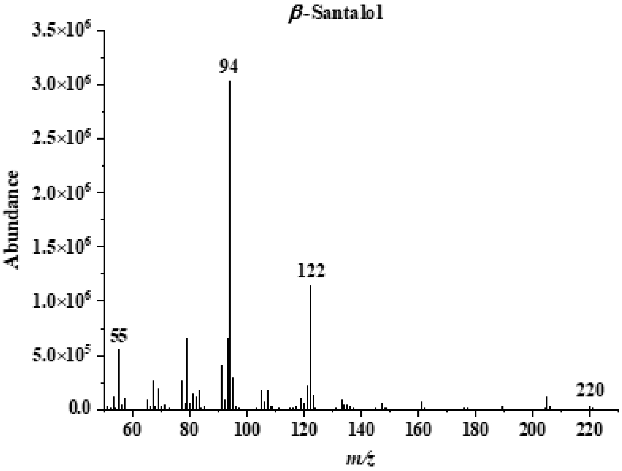
β-Santalol
Abundance
m/z
94
122
55
220

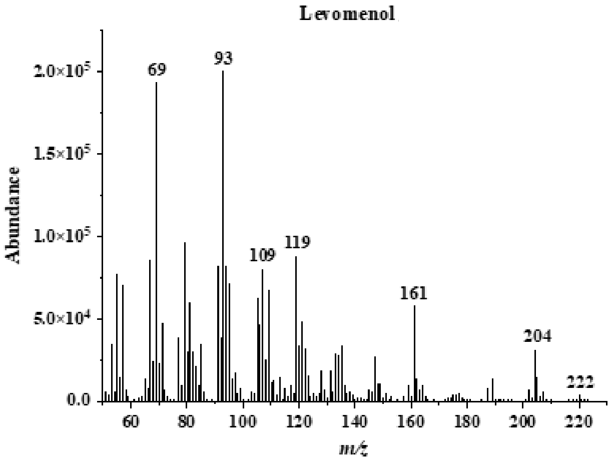
Levomenol
Abundance
m/z
69
93
109
119
161
204
222

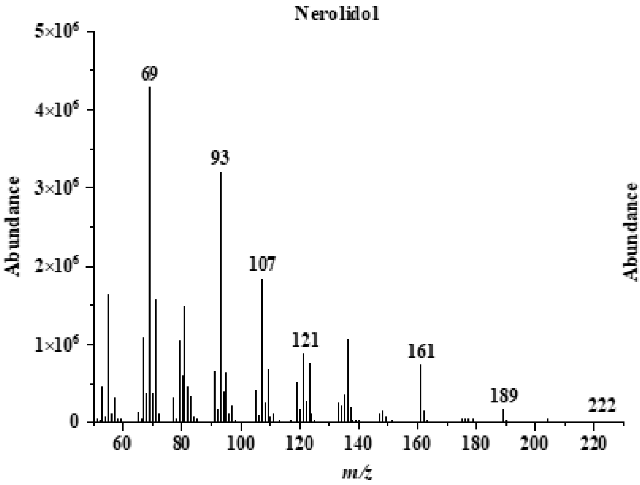
Nerolidol
Abundance
m/z
69
93
107
121
161
189
222

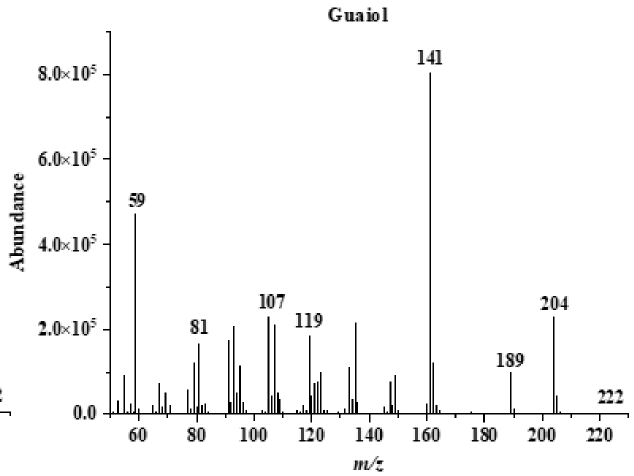
Guaiol
Abundance
m/z
141
59
81
107
119
204
189
222

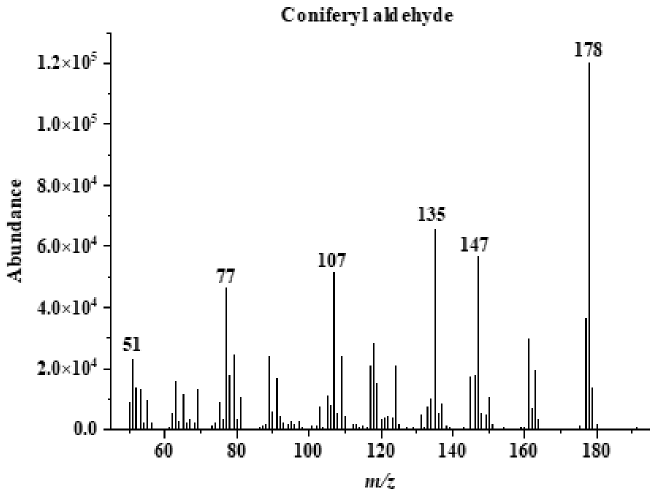
Coniferyl aldehyde
Abundance
m/z
178
135
147
107
77
51

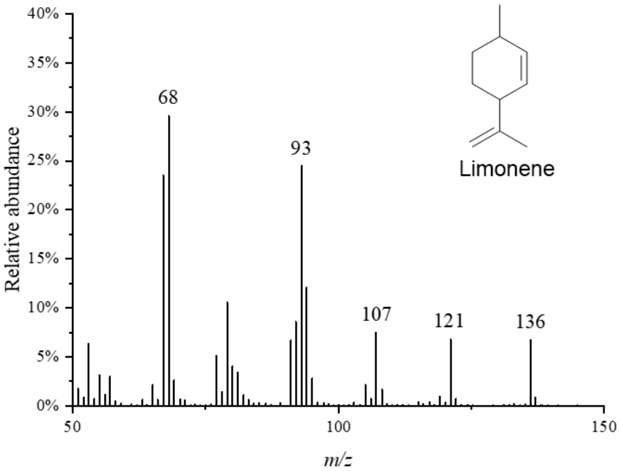
Relative abundance
m/z
Limonene
68
93
107
121
136

MS of peak 1 in figure 7

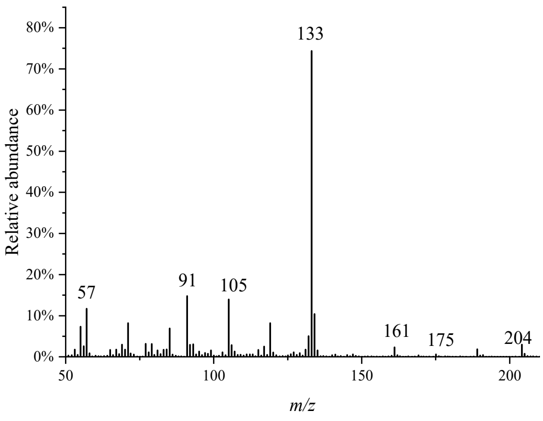

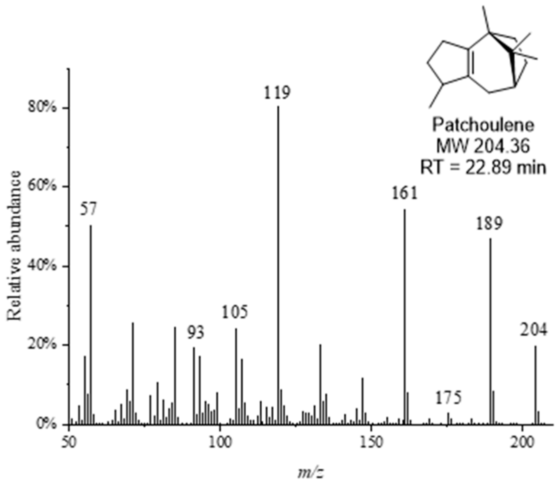

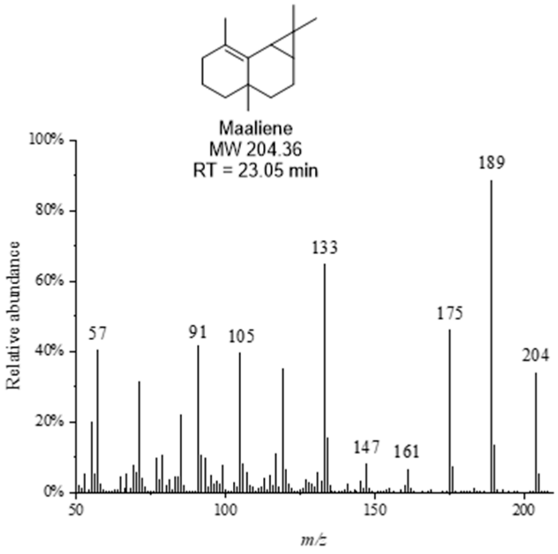

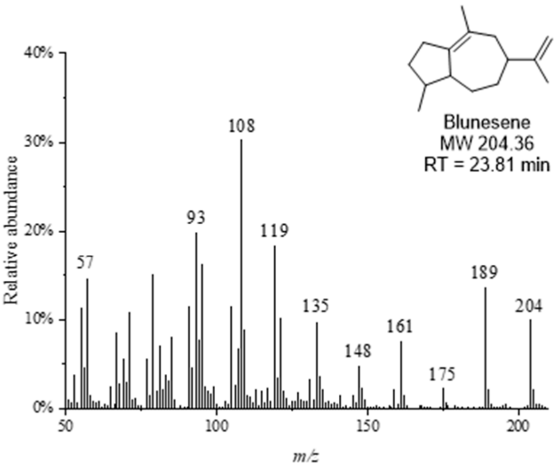

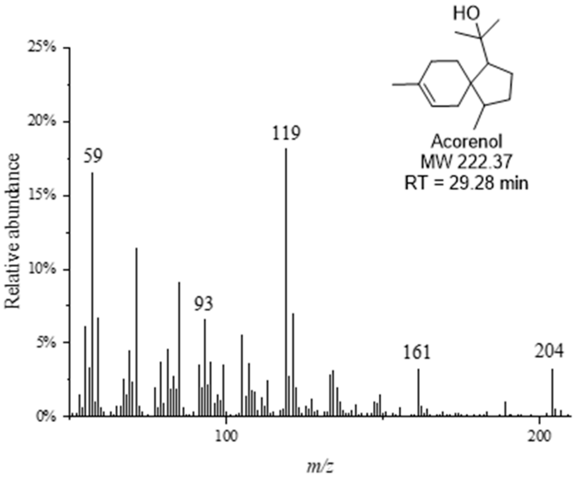

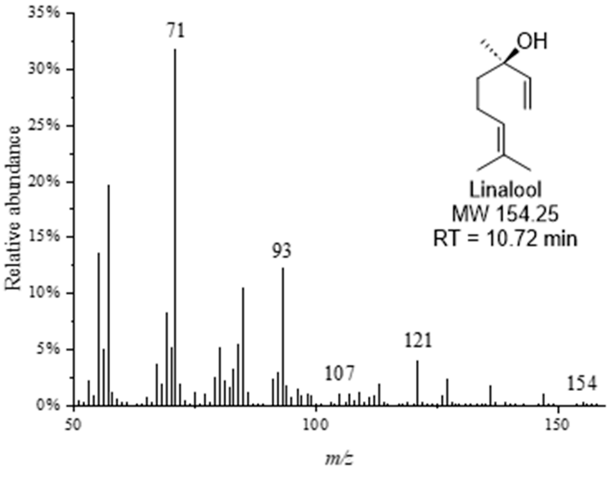

MS of peak 8 in figure 7

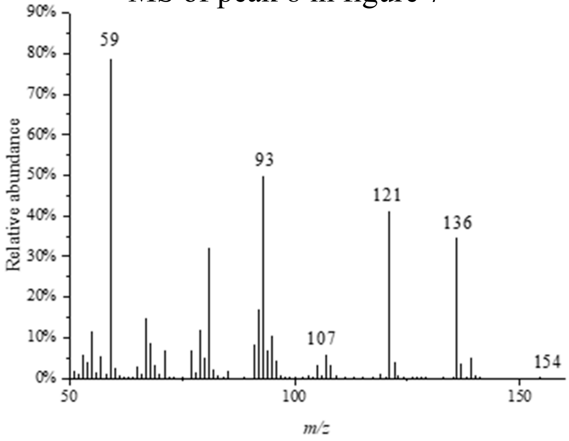

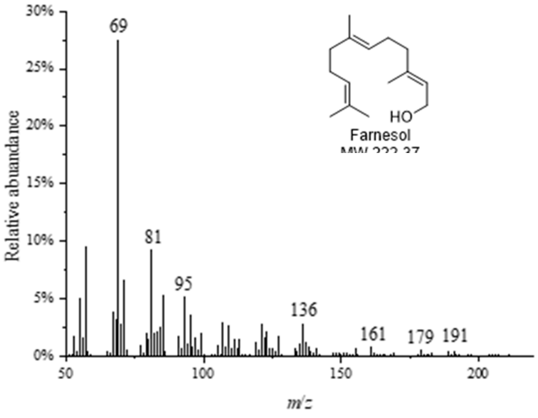

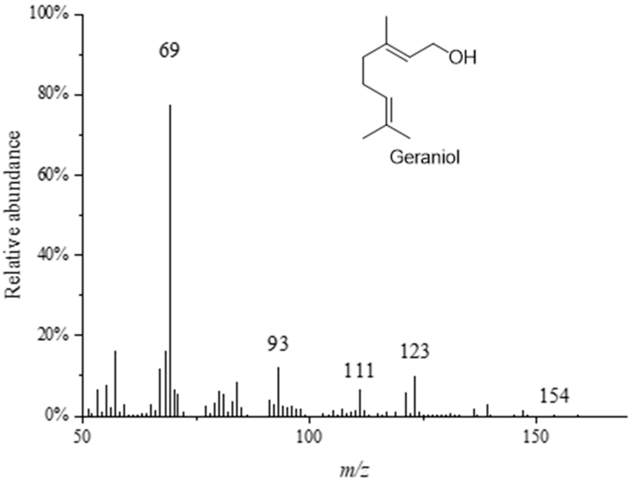

Figure S2. MS spectrum of compounds identified in this study.

Table S1. Primers used in this investigation.

Name	Primer Sequence (5’-3’)
AhTPS1-F	CCATGGCTGATATCGGATCCATGGCTCTTAAACCCTTCT CCCTGTGCACC
AhTPS1-R	TCGAGTGCGGCCGCAAGCTTCTATGTATTGAGGGGTAT GGGGTCCACCAAAAGTG
AhTPS2-F	CCATGGCTGATATCGGATCCATGCAGGCTCTTAAACCC TTCTCCCTGTGC
AhTPS2-R	TCGAGTGCGGCCGCAAGCTTCTATGTATTGAGGAGTAT GGGGTCCACCAAGGTTG
AhTPS3-F	CCATGGCTGATATCGGATCCATGGCATCCCTAGTTTCTC ATGCTACTCCCTTCTCAG
AhTPS3-R	TCGAGTGCGGCCGCAAGCTTTCAAAGCGGAACAAGATC CACCAACAAGGAAGTAATG

Table S2. Retention indices of products generated by the recombinant enzymes.

Identified compounds corresponding to peaks in figure 7	Measured retention indices	Reference retention indices
Unknown sesquiterpene, peak 1	1370	1499 (himachala-2,4-diene)
Patchoulene, peak 2	1426	1432
Maaliene, peak 3	1443	1432
Blunesene, peak 4	1517	1490
Acorenol, peak 5	1599	1598
Limonene, peak 6	1025	1018
Unknown monoterpene, peak 8	1344	1082 (ocimenol)
